# 4-Hydroxy­phenyl 4-fluoro­benzoate

**DOI:** 10.1107/S1600536809000129

**Published:** 2009-01-08

**Authors:** Hyon Pil You, You-Soon Lee, Byung Hee Han, Sung Kwon Kang, Chang Keun Sung

**Affiliations:** a Department of Chemistry, Chungnam National University, Daejeon 305-764, Republic of Korea; b Department of Food Science and Technology, Chungnam National University, Daejeon 305-764, Republic of Korea

## Abstract

In the title compound, C_13_H_9_FO_3_, the dihedral angle between the two benzene rings is 59.86 (4)°. In the crystal, inter­molecular O—H⋯H hydrogen bonds lead to molecular chains propagating in [010].

## Related literature

For general background to whitening agents, see: Ha *et al.* (2007 ▶); Dawley *et al.* (1993 ▶); Nerya *et al.* (2003 ▶); Hong *et al.* (2008 ▶); Lee *et al.* (2007 ▶); Hussain *et al.* (2003 ▶).
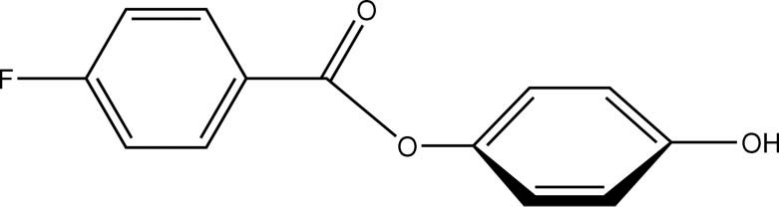


## Experimental

### 

#### Crystal data

C_13_H_9_FO_3_
*M* *_r_* = 232.2
Monoclinic, 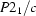

*a* = 24.938 (5) Å
*b* = 5.4789 (11) Å
*c* = 7.6858 (15) Å
β = 93.59 (3)°
*V* = 1048.1 (4) Å^3^
*Z* = 4
Mo *K*α radiation
μ = 0.12 mm^−1^
*T* = 174 (2) K
0.12 × 0.09 × 0.06 mm

#### Data collection

Bruker SMART CCD area-detector diffractometer
Absorption correction: none
10972 measured reflections
2597 independent reflections
2054 reflections with *I* > 2σ(*I*)
*R*_int_ = 0.027

#### Refinement

*R*[*F* ^2^ > 2σ(*F* ^2^)] = 0.041
*wR*(*F* ^2^) = 0.105
*S* = 1.05
2597 reflections
158 parameters
H atoms treated by a mixture of independent and constrained refinement
Δρ_max_ = 0.31 e Å^−3^
Δρ_min_ = −0.24 e Å^−3^

### 

Data collection: *SMART* (Bruker, 2002 ▶); cell refinement: *SAINT* (Bruker, 2002 ▶); data reduction: *SAINT*; program(s) used to solve structure: *SHELXS97* (Sheldrick, 2008 ▶); program(s) used to refine structure: *SHELXL97* (Sheldrick, 2008 ▶); molecular graphics: *ORTEP-3 for Windows* (Farrugia, 1997 ▶); software used to prepare material for publication: *WinGX* (Farrugia, 1999 ▶).

## Supplementary Material

Crystal structure: contains datablocks global, I. DOI: 10.1107/S1600536809000129/bh2214sup1.cif

Structure factors: contains datablocks I. DOI: 10.1107/S1600536809000129/bh2214Isup2.hkl

Additional supplementary materials:  crystallographic information; 3D view; checkCIF report

## Figures and Tables

**Table 1:** Hydrogen-bond geometry (Å, °)

*D*—H⋯*A*	*D*—H	H⋯*A*	*D*⋯*A*	*D*—H⋯*A*
O16—H16⋯O16^i^	0.82 (2)	2.12 (2)	2.9368 (9)	172 (2)

Symmetry code: (i) 
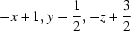
.

**Fig. 1.**
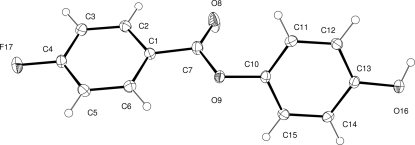
Molecular structure of (I), showing the atom-numbering scheme and 30% probability displacement ellipsoids.

## supplementary crystallographic information

### Comment

Melanin is the pigment responsible for the color of human skin and hair. Tyrosinase is the key enzyme (Ha *et al.*, 2007) that converts tyrosine to melanin and its inhibitors are the target molecules to develop and research anti-pigmentation agents for application to skin. Therefore, treatments using potent inhibitory agents on tyrosinase and melanin formation may be cosmetically useful. Most skin whitening agents currently on the market (Dawley *et al.*, 1993; Nerya *et al.*, 2003) are hydroquinone, ascorbic acid, kojic acid, arbutin, azealic acid, and glycyrrhetinic acid. They contain aromatic, methoxy, hydroxyl (Hong *et al.*, 2008; Lee *et al.*, 2007), or carbonyl functional groups in their structures, and act as a specific functional group to make the skin white by inhibiting the production of melanin. However, most skin whitening agents have some problems, due to toxicity, low stability of formulation and poor skin permeation. In the course of our work on the development of new whitening agents, to complement the inadequacy of current whitening agents and maximize the inhibitory effects of melanin creation, we have synthesized the title compound *via* a general chemical pathway (Hussain *et al.*, 2003) between hydroquinone and 4-fluorobenzoyl chloride.

The 4-fluorobenzoic acid moiety and the 4-hydroxyphenyl ring are essentially planar, with mean deviations of 0.002 and 0.004 Å, respectively, from the corresponding least-squares planes. The dihedral angle between the two benzene rings is 59.86 (4)°. The intermolecular O16—H16···O16*^i^* [symmetry code: (*i*) -*x*+1, *y*-1/2, -*z*+3/2) hydrogen bond allows to form an extensive one-dimensional network, which stabilizes the crystal structure.

### Experimental

Hydroquinone and 4-fluorobenzoyl chloride were purchased from Sigma Chemical Co. and used without further purification. The title compound was prepared from the reaction of 4-fluorobenzoyl chloride (0.159 g, 1 mmol) and hydroquinone (0.132 g, 1.2 mmol) in TEA (8.0 ml). After being stirred for 8 h at 333 K, the mixture was quenched and worked up with ethyl acetate. The mixture was chromatographed on silica gel (2/1 = dichloromethane / ethylacetate) to give the title compound as colorless solid (60%, m.p. 454 K). Single crystals were obtained by slow evaporation of a solution of the title compound in ethyl alcohol and methyl alcohol at room temperature.

### Refinement

Atom H16 of the OH group was located in a difference map and refined freely. Other H atoms were positioned geometrically and refined using a riding model, with C—H = 0.93 Å and with *U*_iso_(H) = 1.2*U*_eq_(carrier C).

### Crystal data

**Table:**

C_13_H_9_FO_3_	*F*(000) = 480
*M**_r_* = 232.2	*D*_x_ = 1.472 Mg m^−^^3^
Monoclinic, *P*2_1_/*c*	Melting point: 454 K
Hall symbol: -P 2ybc	Mo *K*α radiation, λ = 0.71073 Å
*a* = 24.938 (5) Å	Cell parameters from 3698 reflections
*b* = 5.4789 (11) Å	θ = 2.5–28.0°
*c* = 7.6858 (15) Å	µ = 0.12 mm^−^^1^
β = 93.59 (3)°	*T* = 174 K
*V* = 1048.1 (4) Å^3^	Block, colourless
*Z* = 4	0.12 × 0.09 × 0.06 mm

### Data collection

**Table:**

Bruker SMART CCD area-detector diffractometer	*R*_int_ = 0.027
φ and ω scans	θ_max_ = 28.3°, θ_min_ = 0.8°
10972 measured reflections	*h* = −31→33
2597 independent reflections	*k* = −6→7
2054 reflections with *I* > 2σ(*I*)	*l* = −10→10

### Refinement

**Table:**

Refinement on *F*^2^	0 restraints
Least-squares matrix: full	0 constraints
*R*[*F*^2^ > 2σ(*F*^2^)] = 0.041	H atoms treated by a mixture of independent and constrained refinement
*wR*(*F*^2^) = 0.105	*w* = 1/[σ^2^(*F*_o_^2^) + (0.0431*P*)^2^ + 0.436*P*] where *P* = (*F*_o_^2^ + 2*F*_c_^2^)/3
*S* = 1.05	(Δ/σ)_max_ < 0.001
2597 reflections	Δρ_max_ = 0.31 e Å^−^^3^
158 parameters	Δρ_min_ = −0.24 e Å^−^^3^

### Fractional atomic coordinates and isotropic or equivalent isotropic displacement parameters (Å^2^)

**Table:**

	*x*	*y*	*z*	*U*_iso_*/*U*_eq_	
C1	0.17529 (5)	0.4464 (2)	0.47353 (17)	0.0219 (3)	
C2	0.13428 (5)	0.2841 (3)	0.50782 (18)	0.0249 (3)	
H2	0.1423	0.1423	0.5708	0.03*	
C3	0.08163 (6)	0.3318 (3)	0.44905 (19)	0.0275 (3)	
H3	0.0541	0.224	0.4711	0.033*	
C4	0.07154 (5)	0.5443 (3)	0.35679 (18)	0.0270 (3)	
C5	0.11101 (6)	0.7093 (3)	0.32085 (18)	0.0278 (3)	
H5	0.1026	0.8508	0.2581	0.033*	
C6	0.16347 (6)	0.6597 (3)	0.38026 (17)	0.0250 (3)	
H6	0.1908	0.7688	0.3579	0.03*	
C7	0.23063 (5)	0.3826 (3)	0.54191 (18)	0.0259 (3)	
O8	0.24211 (4)	0.2045 (2)	0.62716 (18)	0.0523 (4)	
O9	0.26681 (4)	0.55210 (17)	0.49833 (12)	0.0243 (2)	
C10	0.32036 (5)	0.5229 (2)	0.56439 (16)	0.0207 (3)	
C11	0.35119 (5)	0.3277 (2)	0.51648 (16)	0.0229 (3)	
H11	0.3364	0.2065	0.4436	0.027*	
C12	0.40460 (5)	0.3149 (2)	0.57859 (17)	0.0226 (3)	
H12	0.426	0.1849	0.5474	0.027*	
C13	0.42597 (5)	0.4975 (2)	0.68769 (17)	0.0215 (3)	
C14	0.39464 (5)	0.6938 (2)	0.73353 (17)	0.0234 (3)	
H14	0.4093	0.8157	0.806	0.028*	
C15	0.34135 (5)	0.7073 (2)	0.67088 (17)	0.0225 (3)	
H15	0.32	0.8385	0.7001	0.027*	
O16	0.47887 (4)	0.4939 (2)	0.75328 (14)	0.0302 (3)	
H16	0.4915 (8)	0.356 (4)	0.742 (3)	0.052 (6)*	
F17	0.02021 (3)	0.59316 (18)	0.29800 (13)	0.0424 (3)	

### Atomic displacement parameters (Å^2^)

**Table:**

	*U*^11^	*U*^22^	*U*^33^	*U*^12^	*U*^13^	*U*^23^
C1	0.0203 (6)	0.0223 (7)	0.0231 (6)	0.0016 (5)	0.0006 (5)	−0.0004 (5)
C2	0.0238 (7)	0.0220 (7)	0.0289 (7)	0.0006 (5)	0.0015 (5)	0.0022 (5)
C3	0.0209 (7)	0.0267 (7)	0.0349 (7)	−0.0026 (6)	0.0016 (5)	−0.0021 (6)
C4	0.0198 (7)	0.0289 (7)	0.0317 (7)	0.0047 (6)	−0.0043 (5)	−0.0060 (6)
C5	0.0295 (7)	0.0230 (7)	0.0302 (7)	0.0045 (6)	−0.0035 (5)	0.0010 (5)
C6	0.0249 (7)	0.0227 (7)	0.0273 (7)	−0.0019 (5)	0.0005 (5)	0.0011 (5)
C7	0.0208 (7)	0.0285 (7)	0.0284 (7)	0.0003 (6)	0.0017 (5)	0.0061 (6)
O8	0.0238 (6)	0.0539 (8)	0.0787 (9)	0.0006 (5)	−0.0009 (6)	0.0418 (7)
O9	0.0185 (5)	0.0237 (5)	0.0300 (5)	−0.0015 (4)	−0.0038 (4)	0.0041 (4)
C10	0.0169 (6)	0.0239 (7)	0.0210 (6)	−0.0009 (5)	−0.0015 (5)	0.0036 (5)
C11	0.0255 (7)	0.0206 (7)	0.0224 (6)	−0.0028 (5)	−0.0003 (5)	−0.0013 (5)
C12	0.0225 (7)	0.0202 (6)	0.0252 (6)	0.0020 (5)	0.0024 (5)	−0.0001 (5)
C13	0.0174 (6)	0.0231 (7)	0.0237 (6)	−0.0021 (5)	0.0003 (5)	0.0038 (5)
C14	0.0242 (7)	0.0209 (7)	0.0248 (6)	−0.0032 (5)	−0.0013 (5)	−0.0034 (5)
C15	0.0225 (7)	0.0206 (6)	0.0245 (6)	0.0019 (5)	0.0027 (5)	−0.0012 (5)
O16	0.0186 (5)	0.0288 (6)	0.0423 (6)	0.0007 (4)	−0.0052 (4)	−0.0018 (4)
F17	0.0223 (5)	0.0426 (6)	0.0605 (6)	0.0056 (4)	−0.0106 (4)	0.0019 (5)

### Geometric parameters (Å, °)

**Table:**

C1—C2	1.3928 (19)	O9—C10	1.4078 (15)
C1—C6	1.3932 (18)	C10—C11	1.3806 (19)
C1—C7	1.4872 (18)	C10—C15	1.3824 (18)
C2—C3	1.3863 (19)	C11—C12	1.3881 (18)
C2—H2	0.93	C11—H11	0.93
C3—C4	1.378 (2)	C12—C13	1.3900 (18)
C3—H3	0.93	C12—H12	0.93
C4—F17	1.3572 (15)	C13—O16	1.3826 (16)
C4—C5	1.377 (2)	C13—C14	1.3874 (19)
C5—C6	1.3854 (19)	C14—C15	1.3869 (18)
C5—H5	0.93	C14—H14	0.93
C6—H6	0.93	C15—H15	0.93
C7—O8	1.2000 (17)	O16—H16	0.82 (2)
C7—O9	1.3514 (17)		
			
C2—C1—C6	119.93 (12)	C7—O9—C10	117.74 (10)
C2—C1—C7	117.35 (12)	C11—C10—C15	121.83 (12)
C6—C1—C7	122.72 (12)	C11—C10—O9	121.57 (12)
C3—C2—C1	120.65 (13)	C15—C10—O9	116.47 (12)
C3—C2—H2	119.7	C10—C11—C12	119.06 (12)
C1—C2—H2	119.7	C10—C11—H11	120.5
C4—C3—C2	117.74 (13)	C12—C11—H11	120.5
C4—C3—H3	121.1	C11—C12—C13	119.64 (12)
C2—C3—H3	121.1	C11—C12—H12	120.2
F17—C4—C5	118.34 (13)	C13—C12—H12	120.2
F17—C4—C3	118.43 (13)	O16—C13—C14	117.27 (12)
C5—C4—C3	123.23 (13)	O16—C13—C12	122.04 (12)
C4—C5—C6	118.52 (13)	C14—C13—C12	120.68 (12)
C4—C5—H5	120.7	C15—C14—C13	119.72 (12)
C6—C5—H5	120.7	C15—C14—H14	120.1
C5—C6—C1	119.93 (13)	C13—C14—H14	120.1
C5—C6—H6	120	C10—C15—C14	119.05 (12)
C1—C6—H6	120	C10—C15—H15	120.5
O8—C7—O9	123.63 (13)	C14—C15—H15	120.5
O8—C7—C1	124.63 (13)	C13—O16—H16	109.7 (14)
O9—C7—C1	111.73 (11)		

### Hydrogen-bond geometry (Å, °)

**Table:**

*D*—H···*A*	*D*—H	H···*A*	*D*···*A*	*D*—H···*A*
O16—H16···O16^i^	0.82 (2)	2.12 (2)	2.9368 (9)	172 (2)

Symmetry codes: (i) −*x*+1, *y*−1/2, −*z*+3/2.
